# Unveiling the role of virtual streamer’s interaction attributes in shaping consumer-generated value behaviors

**DOI:** 10.1371/journal.pone.0361335

**Published:** 2026-11-11

**Authors:** Wenjie Li, Guo Cheng, Yun He

**Affiliations:** 1 School of Management Engineering, Chengdu Technological University, Chengdu, Sichuan, China; 2 School of Economics and Business Administration, Yibin University, Yibin, Sichuan, China; 3 School of Business, Sun Yat-sen University, Guangzhou, Guangdong, China; Central China Normal University, CHINA

## Abstract

The interaction attributes of virtual streamers are crucial to their success. This study deconstructs virtual streamers’ interaction into two dimensions: interaction intelligence and interaction humanness. Drawing on immersion theory and parasocial relationship theory, the study examines how these interaction attributes are associated with consumer-generated value behaviors, including social media engagement, donation, and purchase intention. Using data from a survey of 479 respondents and structural equation modeling, the results reveal that both the interaction intelligence and interaction humanness of virtual streamers are positively associated with consumer immersion, which, in turn, is positively related to parasocial relationships with virtual streamers. Immersion and parasocial relationships are further associated with social media engagement, donation, and purchase intention. This study advances the theoretical understanding of virtual streamer interaction and provides practical insights for content creators and platforms aiming to foster consumer-generated value behaviors in virtual streaming.

## 1. Introduction

In recent years, with the rapid development of artificial intelligence (AI) and virtual technologies, the number of virtual streamers has been steadily increasing [1–3], making them a rising force in digital media and marketing communication [4]. An increasing number of companies are adopting virtual streamers in efforts to enhance operational efficiency and improve customer experience [5–7]. With technologies like natural language processing, facial capture, and real-time rendering, virtual streamers can interact with audiences in a highly humanized manner, which may be associated with more immersive experiences [4]. While previous studies have explored the relationships between virtual streamers and consumer attitudes and behaviors, most research remains at a macro level. For example, studies have compared the marketing outcomes associated with human and virtual streamers [7–9], examined attributes such as anthropomorphism, appearance, and personality [10–13], and investigated contextual factors such as system quality, content quality, and entertainment that are related to consumer psychology and behavior [13,14].

However, current discussions on the interaction of virtual streamers remain limited, and the theoretical framework regarding the “interaction attributes” of virtual streamers is still underdeveloped. Existing studies often treat interactivity as one of the fundamental characteristics of virtual streamers, discussing how interactive abilities are related to behaviors such as purchase intention [12,15], viewing intention [13], and brand image [4]. Yet, there is a lack of in-depth analysis of interaction attributes, particularly regarding how intelligence and humanness in interactions are associated with consumer behavior mechanisms. In fact, the interaction of virtual streamers is not solely about “whether they interact,” but more crucially about “how they interact.” Whether consumers perceive virtual streamers as “intelligent,” “emotionally aware,” or “thoughtful” may be associated with the depth of their immersion and emotional connection.

To address this gap, the present study proposes dividing virtual streamers’ interaction attributes into interaction intelligence and interaction humanness. Interaction intelligence refers to a virtual streamer’s ability to quickly and accurately respond to audience inquiries, demonstrating strong semantic understanding and situational adaptation. Interaction humanness refers to a virtual streamer’s ability to perceive and express emotions, showcasing personalized thoughts and intentions, thus being associated with a more humanized interactive experience for consumers. Together, these two dimensions constitute the “intelligence-humanity” duality of virtual streamers’ interaction attributes, offering a new theoretical perspective on understanding the relationship between virtual streamers and consumers. Therefore, this study aims to explore how the interaction attributes of virtual streamers are associated with consumers’ psychological states and behavioral responses.

To comprehensively understand the marketing value of virtual streamers, this study integrates consumer purchase intention, donation, and social media engagement (e.g., likes, comments, shares) into a unified theoretical framework, collectively referring to them as consumer-generated value behaviors. These behaviors reflect the brand and social value created by consumers through various forms of participation within the virtual streamer ecosystem. Additionally, the study focuses on two key psychological mechanisms: immersion and parasocial relationships. The study aims to answer the following questions: (1) How are interaction intelligence and interaction humanness of virtual streamers associated with consumers’ immersion and parasocial relationships? (2) Do immersion and parasocial relationships constitute sequential indirect associations between virtual streamers’ interaction attributes and consumer-generated value behaviors?

This study makes three theoretical contributions. First, by redefining the interaction attributes of virtual streamers in terms of interaction intelligence and interaction humanness, this study enriches the conceptualization of interaction in virtual streamer research [4,12,15]. This classification moves beyond the traditional focus on “interaction frequency” or “perceived interaction intensity”, highlighting the “intelligence-humanity” dual attributes of virtual streamer interactions. Second, by incorporating immersion and parasocial relationships into the research model, this study examines the sequential indirect associations between interaction attributes and consumer behaviors. It highlights the role of immersion in the association between interaction attributes and parasocial relationships, thereby enriching the literature on both immersion [16,17] and parasocial relationships [18–20] within virtual contexts. Finally, by integrating purchase intention, donation, and social media engagement under the unified framework of “consumer-generated value behaviors”, this study expands the research boundaries of consumer value creation and deepens the theoretical explanation of the value co-creation associations in virtual streamer-human interactions [6,10,14,21].

## 2. Theoretical background and literature review

### 2.1. Virtual streamer live streaming

Virtual streamers refer to entities that conduct live streams on social media platforms such as Bilibili, YouTube, and TikTok, using virtual avatars [22]. These streamers typically exhibit anthropomorphic physical and behavioral traits, appearing as 2D or 3D avatars, and can either be operated by real humans or driven by computer algorithms as digital entities [23]. This study primarily focuses on AI-generated virtual streamers, which are digital entities powered by computer algorithms and artificial intelligence [10,24].

In contrast to traditional streamers, virtual streamers, powered by technologies such as motion capture, voice synthesis, or artificial intelligence, are able to provide 24/7 services, with advantages of low cost, low risk, and high interactivity [6,23,25]. In this context, virtual streamers have rapidly become a significant force in the live streaming industry [6]. It has been predicted that by 2030, the virtual streamer market in China will exceed 50 billion RMB, accounting for 40% of the global market, with AI-driven virtual streamers accounting for 30 billion RMB of this total [26].

In recent years, the application of virtual streamers has sparked extensive academic discussion, with researchers comparing the marketing benefits of virtual streamers with those of human streamers [7,9,27,28]. For example, Hu and Ma (2023) [6] noted that the use of sensory language by virtual streamers, due to its lack of authenticity, was associated with lower consumer purchase intention, while sensory language was associated with higher purchase intention for human streamers. Li et al. (2025) [28] found that there are differences between AI streamers and human streamers in sensory, affective, and functional dimensions. Under purchase-oriented motivations, AI streamers with standardized and simple sensory presentation characteristics may be better aligned with consumers’ quick decision-making needs and show advantages over human streamers in functional dimensions. However, under the viewing motivation, human streamers outperform virtual streamers in all three dimensions. Xie et al. (2024) [9] compared the tolerance of consumers for brands following inappropriate comments made by virtual (vs. human) streamers and found that consumers were more tolerant when inappropriate comments were made by virtual streamers. Overall, human streamers excel in emotional expression, spontaneity, and building trust, while virtual streamers are proficient in precise recommendations, personalized services, rapid information processing, and extensive product knowledge [29–31].

Furthermore, many studies have examined virtual streamers as digital entities, analyzing how their attributes are associated with consumer purchase intention, brand image, and other behavioral outcomes [10,13,15]. These studies have explored characteristics such as visual appeal [13], likability, animacy, responsiveness [10], integrity, ability [32], benevolence, predictability [8], anthropomorphism [11,21], sensory language [6], sociality [23], and interactivity [33,34]. For example, Gao et al. (2023) [10] examined the associations between virtual streamers’ likability, animacy, and responsiveness and purchase intention. Xu et al. (2025) [14] found that personalization, human-like personality, system quality, and content quality were positively associated with parasocial interaction and experience value, which were further linked to brand image.

Despite research on the interaction of virtual streamers [4,12,15], most studies have treated it as a unidimensional construct and have not delved deeply into its analysis. This study fills this gap by investigating how the interaction attributes of virtual streamers are associated with the formation of immersion and parasocial relationships, and how these psychological constructs are further related to consumer-generated value behaviors.

### 2.2. Interaction

The interaction of virtual streamers exhibits dual characteristics of both human-computer interaction and interpersonal interaction. The term “interaction” originally referred to interpersonal communication among humans, where it denotes the mutual relationship that arises from exchanges between individuals or groups [35]. With the widespread use of computer technology, the concept of interaction has gradually expanded to include human-computer interaction, encompassing aspects such as bidirectional communication, synchronicity, and active control [4,36,37]. This shift emphasizes the interaction capacity of websites and systems [38] as well as the speed and frequency of interactive responses [39].

Interaction is a core component of live streaming [40]. The interaction in live streaming primarily focuses on whether the streamer can respond quickly and clearly to consumers’ questions, provide relevant product information, and tailor responses to closely align with consumers’ needs [41]. Kang et al. (2021) [42] argued that interaction is associated with greater consumer intimacy and engagement during live streams. Zhou & Tian (2022) [43] noted that a streamer’s interaction is related to streaming effectiveness and sales. Liu et al. (2022) [40] suggested that interaction is significantly related to consumers’ flow experiences and trust, which are further associated with purchase intention. Shiu et al. (2023) [44] emphasized that interaction in live streaming is positively related to immersion experiences and is also related to purchase intention. Previous studies have mostly treated interaction as a single dimension, with some differentiating it based on the interaction objects, categorizing it into viewer-streamer interaction and viewer-viewer interaction [45].

For virtual streamers, interaction in live streaming is an important aspect of their commercial success [12]. The interaction of virtual streamers not only pertains to information transmission, emotional connection, and the maintenance of social interactions [12] but is also related to consumers’ loyalty, trust, and purchase intention [4,46]. As non-human entities, virtual streamers’ interactions distinctly exhibit dual characteristics of both human-computer interaction and interpersonal interaction. From the perspective of human-computer interaction, virtual streamers’ interaction is expected to be intelligent, meaning they should be able to respond quickly and effectively to audience needs. From the perspective of interpersonal interaction, virtual streamers must also exhibit humanness, meaning they should be able to perceive and express emotions, showcasing personalized thoughts and intentions. Shortcomings in intelligence and warmth in virtual streamers may be associated with less favorable consumer experiences.

In human-computer interaction, humans expect non-human entities to meet their cognitive and emotional needs, handle diverse tasks ranging from cognitive analysis to emotional socialization, understand and respond to consumers’ emotions, and provide a sense of companionship [47,48]. Additionally, according to the theory of anthropomorphism, human traits are typically embodied in appearance, behavior, and thought [49,50]. As highly anthropomorphized non-human entities, virtual streamers are often expected to exhibit anthropomorphic characteristics in all three aspects: (1) appearance that resembles a human; (2) actions that are vivid and natural; and (3) the ability to interact with consumers in a human-like manner [49,51,52]. Interaction, as a primary manifestation of virtual streamers’ behavior, includes not only efficient interaction capability (interaction intelligence) but also higher-level, humanized interactivity (interaction humanness).

However, previous studies on virtual streamers have often treated interaction as a basic trait and have discussed it as a unidimensional construct, lacking in-depth analysis and mostly focusing on whether virtual streamers can respond promptly to consumer comments. These studies rarely address the emotions and thoughts conveyed during the interaction or consider the humanizing aspects of interaction [33,34]. For instance, Na et al. (2025) [33] proposed that perceived interaction refers to “consumers’ perception of whether virtual streamers can respond quickly and flexibly through human-like features, such as facial expressions, voice, and body language.” Liu et al. (2025) [53] categorized the interaction between virtual streamers and consumers into product interaction and social interaction.

This study focuses on the distinctiveness of virtual streamers by deconstructing their interaction attributes into two dimensions: (1) interaction intelligence, which refers to the virtual streamer’s ability to understand consumers’ expressions and needs, promptly respond to questions, and provide relevant, reasonable answers; and (2) interaction humanness, which refers to the virtual streamer’s ability to perceive and express emotions and demonstrate their thoughts or intentions. The former represents the basic level of interaction, reflecting the virtual streamer’s understanding of audience needs and task-handling ability, while the latter represents a higher level of interaction, showcasing the virtual streamer’s more human-like attributes, including autonomous thought and emotional expression. This deconstruction not only reflects the dual identity of virtual streamers as both technological entities and socialized others but also provides a new theoretical framework for understanding how their interaction attributes are related to consumer-generated value behaviors.

### 2.3. Immersion

Immersion refers to a psychological state in which consumers are fully engaged in a particular domain, to the point of losing self-awareness. Its defining characteristic is the feeling of being surrounded and enveloped by an experience [16,17]. Extensive research has explored the associations involving immersion across various domains, including gaming, virtual reality (VR), augmented reality (AR), tourism, and live streaming [54].

In the context of live streaming, immersion is defined as “the state in which viewers feel attracted, engaged, and immersed in the live stream” [55]. Immersion has been widely examined in relation to consumer participation and positive consumer behaviors. For instance, Hewei (2022) [56] suggested that high levels of immersion are associated with stronger consumer purchase intention for apparel in short-form videos. Guan et al. (2022) [57] found that immersion is related to consumer purchase intention during live streaming. Sun et al. (2019) [58] proposed that immersion is also associated with consumers’ willingness to send virtual gifts. Qin and Liu (2025) [34] highlighted that immersive experiences provided by live streaming platforms are associated with consumers’ willingness to send gifts and engage in interactive behaviors.

Many studies have explored the correlates and related factors of immersion, but there has been limited analysis of the in-depth relationship between interaction attributes and immersion. For example, Sun et al. (2019) [58] identified technological features in live streaming, such as visibility, metavoicing, and guided shopping, that are related to immersion. Guan et al. (2022) [57] suggested that responsiveness, self-presentation, social orientation, and harmony in live streaming are positively associated with immersion in relation to the perceived proximity and sense of belonging between viewers and streamers. Hewei (2022) [56] argued that media interaction and perceived value in mobile short video applications are also associated with immersive experiences. Li and Sun (2025) [59] proposed that perceived interaction in interactive video content is associated with immersion and empathy, which in turn could be linked to arousal and purchase intention.

In the research on virtual streamers, some scholars have also explored issues related to immersion. For example, Lee and Lee (2023) [60] suggested that platform type and the perceived role of the virtual streamer are related to viewers’ sense of presence and immersion. Based on consistency theory, Chen et al. (2025) [61] proposed that the streamer’s persona-live content congruence, the viewer’s interest-live content congruence, and value congruence between viewer and streamer are associated with immersion, which is further associated with behavioral intention. However, no study has yet positioned “immersion” as a psychological linking variable between virtual streamers’ interaction attributes and consumer-generated value behaviors.

This study, based on the interaction attributes of virtual streamers, aims to explore the indirect linking role of immersion in the relationship between interaction intelligence, interaction humanness, and consumer-generated value behaviors.

### 2.4. Parasocial relationships

People often develop one-sided, “abnormal social relationships” with “target objects” that cannot respond to their emotions, which may be related to their complex psychological needs [62]. This type of relationship is known as a parasocial relationship [18–20]. Due to the unprecedented interactivity of social media, influencers on these platforms are able to establish close relationships with their followers [63]. As a result, parasocial relationships are frequently used to understand how people form relationships with media figures such as celebrities, influencers, and streamers, and the behaviors associated with those relationships [45].

Previous studies have explored the attributes of influencers, such as attractiveness, similarity, and expertise [63,64], as well as technological environment characteristics, such as information quality, system quality, and design quality, and their associations with parasocial relationships [65]. Additionally, research has examined the role of parasocial relationships in marketing outcomes, including brand interest, brand attitude, engagement, purchase intention, and brand loyalty [66–69].

The real-time interactivity of live streaming enables streamers to better engage with consumers, and such engagement is often associated with the development of parasocial relationships [68]. Behaviors such as self-disclosure and emotional expression during interactions are also associated with parasocial relationships and mutual understanding [45,70]. In live streaming, positive interactions are related to greater intimacy between consumers and streamers, and viewers may report a sense of familiarity with the streamer [57]. However, despite occasional responses from the streamer to audience interactions, they cannot respond to every interaction, nor can they establish genuine friendships with every viewer. Therefore, the interaction remains inherently one-sided and parasocial [71,72].

Although virtual streamers are not real humans, based on the Computers Are Social Actors (CASA), consumers still develop parasocial relationships with virtual streamers [73]. Establishing parasocial relationships is often discussed as an important strategy employed by virtual streamers during live streams [74]. Consumers who report a parasocial relationship with a virtual streamer also tend to report higher satisfaction and stronger willingness to continue watching the stream [75]. Some studies suggest that virtual streamers are particularly adept at building parasocial relationships through live streaming platforms, which is associated with a sense of closeness [74]. However, other research has found that compared to human streamers, the parasocial relationships formed with humanoid virtual influencers are weaker, and consumer brand interest and purchase intention are lower [69]. This study argues that the deep interaction provided by virtual streamers is likely to be associated with stronger parasocial relationships. Consumers who report a stronger parasocial relationship with a virtual streamer may be more likely to purchase products recommended by the streamer and actively engage in donation behavior.

### 2.5. Consumer-generated value behaviors

Businesses can derive diversified value from consumers, including monetary value gained directly through consumer purchases of products or services, as well as indirect value obtained through behaviors such as social media engagement (“like”, “comment”, “repost”, etc.) and recommending products to others [76,77]. In the live streaming context, virtual streamers can obtain “traffic” value through engagement behaviors such as likes, comments, and shares, “virtual currency” value through donations, and “product promotion revenue” through the purchase of recommended products. However, Lou et al. (2023) [78] pointed out that although virtual influencers have shown favorable outcomes in relation to brand image and brand awareness, their persuasiveness in relation to purchase intention remains limited in contexts involving low authenticity, low similarity with fans, and weak parasocial relationships with consumers. This study adopts a comprehensive perspective of value generation behavior to explore three types of consumer-generated value behaviors in the virtual streamer live streaming context: purchase intention, donation, and social media engagement.

Purchase intention refers to consumers’ intent to buy products recommended by virtual streamers. Previous research has frequently explored consumer behaviors related to purchasing products recommended by virtual streamers [10,12,33,53]. For example, Gao et al. (2023) [10] found that characteristics such as the likeability, animacy, and responsiveness of virtual streamers are positively associated with consumer purchase intention, with social presence and telepresence serving as indirect linking variables. Gu et al. (2025) [15] highlighted that key traits of virtual streamers, such as perceived authenticity, anthropomorphism, social presence, and interactivity, are positively associated with purchase intention through their relationships with attractiveness and perceived cognitive fluency. Qin and Liu (2025) [34] also suggested that virtual streamers’ perceived competence, interaction quality, and warmth are associated with purchase intention through their relationships with transactional psychological contact and relational psychological contracts.

In addition to purchasing behavior, consumers actively engage in streamers’ activities such as liking, commenting, sharing, and saving content. While these behaviors do not directly generate income, they are related to the streamer’s “traffic” and are crucial for virtual streamers. Research on virtual streamers has mainly focused on sustained viewing behaviors, with relatively few studies on other forms of social media engagement. For instance, Chen et al. (2025) [61] used consistency theory to discuss the relationships between congruence between streamers’ personas and live content, as well as viewers’ interests and values, and sustained viewing behavior.

Donation behavior is another key source of revenue for live streaming platforms and content creators [79]. Donation refers to consumers voluntarily sending virtual gifts or making direct monetary transfers without expecting a return [80,81]. Unlike online shopping, donating virtual gifts in live streaming is a voluntary act [45]. In live streaming, viewers perceive donating as a means of obtaining emotional value, visibility, or perceived closeness, and donation is often associated with a stronger perceived bond with the streamer and symbolic recognition [80]. Donation behavior, as a form of gifting, has been shown to be associated with the establishment or maintenance of a social exchange relationship with the recipient [82]. Lee et al. (2025) [4] explored the relationships among virtual streamers’ interaction, consumers’ proactive engagement behaviors, such as sharing emotions and expressing feelings, and immediate donation intentions. Chen et al. (2025) [61] discussed the relationships among streamer persona-live content congruence, viewer interest-live content congruence, viewer-streamer value congruence, and gift-giving behavior.

Overall, research on virtual streamers has primarily focused on consumers’ purchasing behaviors [8,10–12,15,53], with relatively fewer studies on social media engagement and donation behaviors [83]. This study integrates these three parallel behaviors comprehensively under the term “consumer-generated value behaviors” and explores the relationships between virtual streamers’ interaction attributes and each specific behavior, as well as the indirect association pathways underlying these relationships.

## 3. Hypothesis development

### 3.1. Interaction intelligence

#### 3.1.1. The relationship between interaction intelligence and immersion.

The interaction intelligence of virtual streamers refers to their ability to understand the audience’s expressions and needs, respond promptly and accurately, and provide relevant and reasonable answers, while being perceived as intelligent and competent. Previous studies have indicated that positive interaction is associated with a stronger audience sense of social presence and immersion [55,84]. When interacting with virtual beings, consumers are more likely to trust those who demonstrate high levels of intelligence, such as virtual agents capable of understanding, automating, and personalizing responses [85]. Furthermore, smarter virtual beings are significantly associated with greater consumer trust and satisfaction [86].

In the live-streaming context, when consumers pose questions to a virtual streamer, an intelligent virtual streamer can quickly and efficiently respond to the questions, understand and meet the consumers’ personalized needs, and provide reasonable solutions. This process may be associated with lower cognitive load, greater interaction fluency, and stronger immersive experiences. Fang et al. (2018) [87] noted that when consumers are able to smoothly address their needs during interactions with virtual streamers, they tend to report stronger immersion. Effective interaction would be associated with a greater likelihood of experiencing immersion and deep engagement [4,88]. Therefore, we propose the following hypothesis:

**H1a:** Interaction intelligence is positively associated with immersion.

#### 3.1.2. The relationship between interaction intelligence and parasocial relationships.

In online environments, interaction has been discussed as an important factor related to maintaining relationships [89]. Previous studies have found that influencers with strong interaction skills are associated with stronger parasocial relationships among consumers [90]. Virtual streamers engage with consumers through comments, greetings, and real-time responses, which are associated with psychological connections and trust [4,33,91]. Moreover, intelligent virtual streamers can analyze big data to quickly understand and respond to consumers’ needs, providing personalized feedback, suggestions, and solutions. This type of interaction is associated with consumers feeling cared for and valued [92], as well as narrower psychological distance between virtual streamers and consumers [93] and stronger emotional connections [94,95]. At the same time, virtual streamers can provide appropriate responses based on consumers’ emotions, which may be associated with consumers’ feelings of being understood and with higher perceived empathy toward the streamer.

Research has shown that when virtual streamers are perceived as able to understand consumers’ implicit needs, consumers tend to report more favorable shopping experiences, while less intelligent virtual streamers may be associated with consumer dissatisfaction [96]. These positive interactive experiences are associated with the establishment of parasocial relationships. Based on this, we propose the following hypothesis:

**H1b:** Interaction intelligence is positively associated with parasocial relationships.

### 3.2. Interaction humanness

#### 3.2.1. The relationship between interaction humanness and immersion.

The interaction humanness of virtual streamers refers to their ability to possess independent thoughts, perceive and empathize with the consumer’s emotions, and express their own genuine feelings during real-time interactions. This construct differs from the general anthropomorphism perception. General anthropomorphism typically refers to the attribution of human-like surface features to non-human entities, primarily focusing on visual and auditory resemblance (e.g., human-like avatars, voices, or physical movements) [49]. In contrast, interaction humanness in live streaming goes beyond superficial resemblance. It captures deep-level “mind perception” during reciprocal communication. When engaging with consumers, virtual streamers endowed with interaction humanness not only recognize feedback and respond with appropriate emotions (e.g., happiness, sadness, or disappointment), but also initiate conversations and express unique perspectives, rather than passively answering questions or simply catering to consumers’ preferences. For instance, if a viewer criticizes the virtual streamer, the streamer can understand the negative feedback and respond with emotional resonance.

This study posits that such deep-level interaction humanness is an important factor associated with consumer immersion. Rather than relying on mere visual realism [97], interaction humanness is associated with immersion in relation to emotional realism and social presence. When virtual streamers display their own thoughts and empathetic emotions, consumers are more likely to perceive them as social actors rather than programmed machines [98]. This profound sense of social connection is associated with consumers’ interest and curiosity, as well as with a deeply absorbing psychological state [99]. Furthermore, drawing on the humanness-value-loyalty model [100], the deep humanness traits, i.e., emotional empathy and autonomous thought, of virtual streamers are associated with higher perceived social and emotional value among consumers. Positive perceived value has also been associated with greater consumer immersion during the service encounter [56]. Therefore, we propose the following hypothesis:

**H2a:** Interaction humanness is positively associated with immersion.

#### 3.2.2. The relationship between interaction humanness and parasocial relationships.

For virtual streamers, possessing only interaction intelligence is often associated with more limited deeper and more thoughtful exchanges with consumers, as well as weaker parasocial relationships. Perceived anthropomorphism is considered an important factor associated with the formation of parasocial relationships [67]. Therefore, virtual streamers who resonate emotionally and demonstrate high levels of anthropomorphism in terms of thoughts and intentions may be associated with stronger consumer empathy and greater expectations of long-term interactions in the future [101]. Although many AI-driven virtual streamers have achieved high levels of intelligence, there remains a gap in their humanlike qualities.

When consumers perceive virtual streamers to possess strong human-like attributes, they often find them more engaging. This perception of being “engaging” is associated with consumers’ positive interactive attitudes and stronger expectations of continued interaction with the virtual streamer [102]. Thus, the interaction humanness of the virtual streamer may be positively associated with the development of parasocial relationships with consumers. Based on this, we propose the following hypothesis:

**H2b:** Interaction humanness is positively associated with parasocial relationships.

### 3.3. Immersion

#### 3.3.1. The relationship between immersion and parasocial relationships.

From a relationship formation perspective, a deep psychological connection with a virtual persona is often discussed in relation to an initial state of psychological engagement. We posit that immersion is associated with this initial state. Immersion is associated with consumers’ investment of substantial cognitive resources into their interactions with virtual streamers, as well as with greater familiarity, intimacy, and parasocial relationships [103].

Beyond cognitive processing, immersion theory indicates that immersive experiences are closely associated with emotional involvement [104]. When consumers are deeply absorbed in a streamer’s interactive context, they tend to report stronger emotional attachments and stronger perceptions of the streamer as a friend or acquaintance [105]. In live-streaming environments, this psychological absorption is associated with a stronger sense of presence among viewers. Even if interactions are system-simulated or one-sided, such absorption may be linked to a smaller perceived distance between the viewer and the virtual streamer, as well as to a more ongoing relational connection.

Finally, high levels of immersion are positively associated with heightened perceived realism and anthropomorphic qualities of virtual beings, both of which are established correlates of parasocial relationships [67]. These combined cognitive, emotional, and perceptual association pathways suggest that the state of being immersed is positively related to stronger parasocial connections. Thus, we propose:

**H3a:** Immersion is positively associated with parasocial relationships.

#### 3.3.2. The relationship between immersion and consumer-generated value behavior.

Previous research has shown that immersion is associated with positive emotions and memorable experiences [106], while also being related to viewers’ behavioral intentions [34]. Additionally, studies have indicated that when customers are immersed in an experience, they are more likely to recognize the value and benefits they can gain from live streaming, and such recognition is associated with purchasing decisions and engagement behaviors [87].

In the context of virtual streamers, the positive interaction provided is associated with consumers’ perception that they are interacting with a real person, as well as with a stronger sense of immersion. This immersion is often accompanied by enjoyment and pleasure among the audience, which have been linked to positive attitudes [61] and could be positively associated with consumer-generated value behaviors [58,107], such as purchasing products recommended by the streamer, actively engaging in the streamer’s activities, and donating to the streamer [61]. Therefore, we propose the following hypotheses:

**H3b:** Immersion is positively associated with social media engagement.
**H3c:** Immersion is positively associated with donation.
**H3d:** Immersion is positively associated with purchase intention.

### 3.4. The relationship between parasocial relationships and consumer-generated value behavior

Previous research has shown that parasocial relationships are associated with greater trust in the streamer or influencer, and such trust is related to positive attitudes toward products and purchase intention [108]. Additionally, parasocial relationships are associated with positive emotional responses in viewers, such as enjoyment, loyalty, trust, and satisfaction, which could further be related to behaviors such as donation and purchasing [80].

Furthermore, the sense of intimacy and parasocial relationships established with virtual streamers are associated with consumer decisions based more on social, emotional, and symbolic values, rather than solely on utilitarian value. In this context, consumers tend to report stronger psychological and behavioral support for virtual streamers, rather than merely engaging in a transactional relationship with them [80]. Therefore, we hypothesize that consumers who report stronger parasocial relationships with virtual streamers and perceive them as friends also tend to report stronger purchase intention, donation intention, and engagement intention. Based on this, we propose the following hypotheses:

**H4a:** Parasocial relationships are positively associated with social media engagement.
**H4b:** Parasocial relationships are positively associated with donation.
**H4c:** Parasocial relationships are positively associated with purchase intention.

Based on the hypotheses developed above, the theoretical framework for this study is presented in Fig 1.

**Fig 1:**
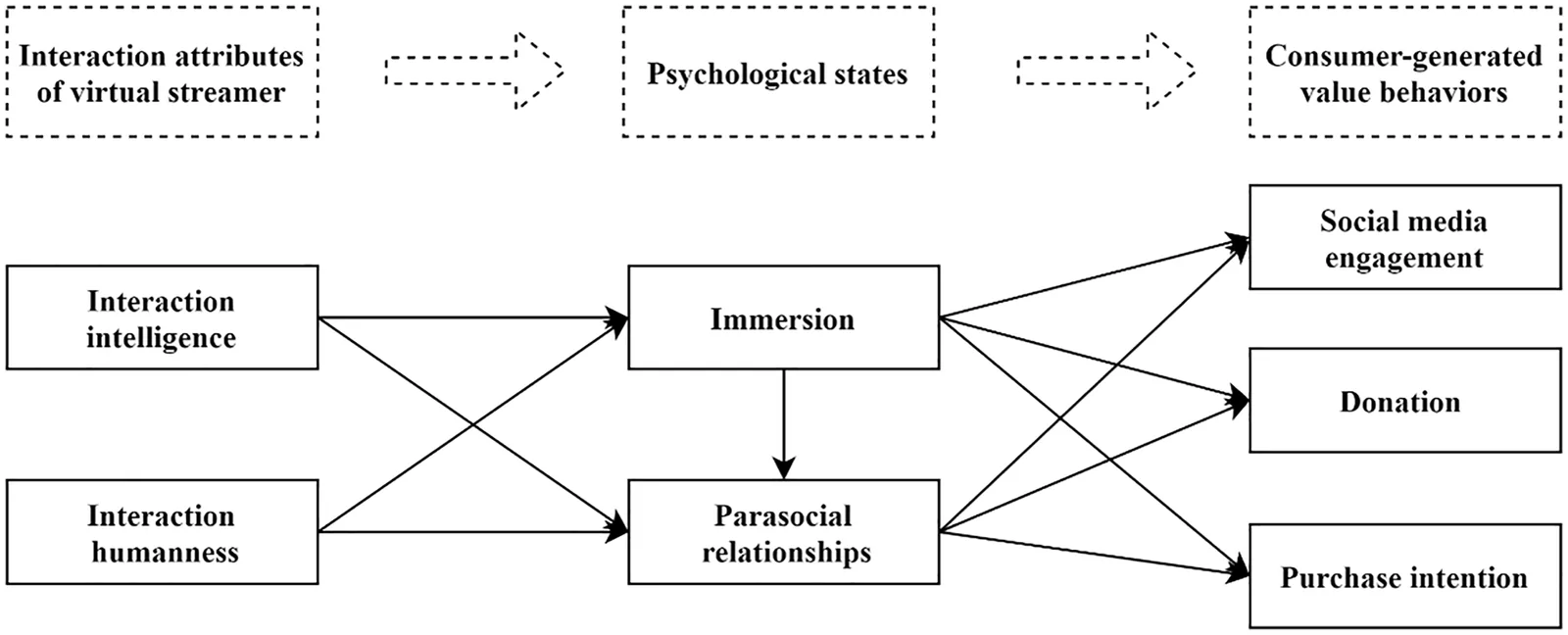
Research theoretical framework.

## 4. Research methodology

### 4.1. Measurement

All measurement items were adapted from prior literature and modified to suit the context of virtual streamers. Specifically, four items measuring interaction intelligence were adapted from W. Gao et al. (2023) and Moussawi et al. (2021) [10,109]. Similarly, four items for interaction humanness and three for immersion were derived from [52] and [58], respectively. Four items for parasocial relationships were adapted from Cheung et al. (2022) and Farivar et al. (2021) [65,110]. Furthermore, six items for social media engagement were adapted from He et al. (2022) and Raza et al. (2023) [111,112]; three items for donation were adapted from Chou & Nguyen (2023) and H. Lee et al. (2025) [4,82]; and three items for purchase intention were adapted from [10]. To ensure response quality and mitigate potential response set bias, one item measuring purchase intention (PI3) was reverse-coded prior to data analysis. All items were measured on a 7-point Likert scale.

### 4.2. Data collection process

An online survey was administered to empirically test the research hypotheses proposed in this study. Credamo (http://www.credamo.com/) was selected as the platform for creating and distributing the online survey. As a widely recognized professional survey platform in China, Credamo has proven to be a highly reliable tool for academic data collection [111]. One notable advantage of Credamo is that it provides access to a broad and diverse non-student demographic, which aligns perfectly with this study’s research objectives. This study involving human participants was reviewed and approved by the Ethics Committee of the School of Management Engineering, Chengdu Technological University (Approval No. ME2026001). Data collection commenced only after ethics approval had been obtained and was carried out from January 7, 2026 to January 20, 2026. Electronic informed consent was obtained from all participants before they completed the online questionnaire, and all responses were collected anonymously to protect participant privacy.

A total of 525 questionnaires were collected, yielding 479 valid responses after excluding respondents who failed the attention checks or provided identical responses across all survey items. Because the online survey was configured to require mandatory answers for all items, there were no missing values in the final dataset. Descriptive statistics of the respondents are presented in Table 1. The majority of respondents (78.2%) were between 21 and 35 years old, and 46.1% were male respondents. The adequacy of the sample size was assessed using established PLS-SEM guidelines [113]. According to the widely accepted “10-times rule” heuristic, the minimum sample size should be ten times the maximum number of structural paths pointing at a particular latent construct. In our model, a maximum of five predictors including control variables point to a single endogenous construct, yielding a minimum requirement of 50 responses. The final sample of 479 respondents substantially exceeds this heuristic minimum and is adequate for estimating the proposed model.

**Table 1:** Demographic information of respondents (n = 479).

Profile	Characteristics	Frequency	Percentage
Age	<=20	26	5.4%
	21-24	108	22.5%
	25-30	153	31.9%
	31-35	114	23.8%
	36-40	44	9.2%
	41-50	23	4.8%
	51-60	11	2.3%
	>60	0	0
Gender	Male	221	46.1%
	Female	258	53.9%
Education	Primary School	0	0%
	Middle School	2	0.4%
	High School	14	2.9%
	Bachelor’s degree/Junior college diploma	384	80.2%
	Master’s degree	73	15.2%
	Doctoral degree	6	1.3%

## 5. Data analysis

In this study, data analysis was conducted following a standard two-stage approach [114]. We employed SmartPLS 4.0 to evaluate both the measurement and structural models, as Partial Least Squares Structural Equation Modeling (PLS-SEM) is particularly well-suited for complex models and exploratory research. Specifically, the PLS algorithm was executed using the path weighting scheme, a maximum of 3,000 iterations, and a stop criterion of 10^−7^ to ensure rigorous algorithm convergence. Furthermore, a non-parametric bootstrapping procedure with 5,000 resamples was applied to assess the statistical significance of all measurement model parameters, structural paths, and specific indirect associations. Additionally, IBM SPSS Statistics 27.0 was utilized for preliminary data screening, descriptive statistics, and testing for common method bias (CMB).

### 5.1. Measurement model

The measurement model was assessed by evaluating the reliability and validity of the constructs. First, we calculated Cronbach’s α (CA), rho_A, composite reliability (CR), average variance extracted (AVE), and item loadings to evaluate the constructs’ internal reliability and convergent validity (see Table 2).

**Table 2:** Reliability and validity results.

Variables	Cronbach’s Alpha	rho_A	CR	AVE	Factor Loadings
Interaction Intelligence [10,109]	0.806	0.820	0.873	0.633	
II1: Her/His responses are closely related to my questions.					0.855
II2: She/He is capable of communicating and interacting with humans.					0.754
II3: She/He can respond to my questions promptly.					0.824
II4: She/He is intelligent.					0.743
Interaction humanness [52]	0.933	0.938	0.952	0.833	
IH1: She/He can experience emotions.					0.906
IH2: She/He has a mind of his/her own.					0.919
IH 3: She/He has intention.					0.916
IH 4: She/He has free will.					0.910
Immersion [58]	0.734	0.738	0.850	0.654	
IM1: I feel fully absorbed.					0.859
IM2: My attention is deeply captivated.					0.773
IM3: When watching her/his livestreams or videos, or when communicating or interacting with her/him, my attention is highly focused.					0.792
Parasocial relationships [65,110]	0.866	0.868	0.909	0.713	
PSR1: I feel a sense of closeness with her/him.					0.826
PSR2: Overall, I feel like I am friends with her/him.					0.879
PSR3: I feel like she/he is an old friend of mine.					0.848
PSR4: I feel like I am a part of the group she/he belongs to.					0.824
Social media Engagement [111,112]	0.848	0.850	0.888	0.569	
SME1: I would comment or send bullet chats to interact with her/him.					0.772
SME2: I would follow her/his social media accounts.					0.710
SME3: I am willing to join her/his fan community.					0.812
SME4: I would bookmark or save the content she/he posts.					0.777
SME5: I would like the content she/he posts.					0.714
SME6: I would share or repost the content she/he publishes.					0.736
Donation [4,82]	0.909	0.914	0.943	0.846	
DN1: I am willing to pay or spend money to watch the content she/he creates.					0.922
DN2: I would give her/him tips or donations.					0.905
DN3: I would purchase and send her/him virtual gifts.					0.933
Purchase intention [10]	0.907	0.914	0.941	0.843	
PI1: I am willing to purchase products she/he recommends.					0.934
PI2: I expect to purchase the products she/he recommends.					0.921
PI3: I do not intend to purchase the products she/he recommends. (Reversed-coded)					0.899

The results demonstrated robust internal consistency, with all constructs’ Cronbach’s α (CA) and composite reliability (CR) scores exceeding the ideal value of 0.7 [115]. Similarly, the scores of rho_A ranged from 0.738 to 0.938, surpassing the threshold value of 0.7 [116]. The AVE values of all of the constructs ranged from 0.569 to 0.846, thus exceeding the suggested threshold of 0.5 [115]. In addition, the item loadings for the majority of the constructs exceeded the 0.7 benchmark.

We then evaluated discriminant validity using three distinct methods. First, the cross-loadings criterion revealed that all constructs’ item loadings loaded most strongly on their intended constructs and not on others. Second, in accordance with the Fornell-Larcker criterion, all values of the square root of the AVE were larger than any correlation coefficient between constructs, suggesting good discriminant validity (see Table 3). Finally, the largest HTMT ratio was 0.806, which is less than the stringent threshold of 0.85 [117] (refer to Table 4). In summary, these results suggest that our measurement model exhibits sufficient reliability and validity.

**Table 3:** Correlation coefficient matrix and square roots of AVEs (Fornell-Larcker).

	SME	DN	IH	IM	II	PSR	PI
**SME**	0.754						
**DN**	0.694	0.920					
**IH**	0.367	0.516	0.913				
**IM**	0.581	0.547	0.387	0.809			
**II**	0.479	0.453	0.540	0.485	0.795		
**PSR**	0.692	0.633	0.544	0.580	0.534	0.845	
**PI**	0.639	0.653	0.432	0.527	0.451	0.619	0.918

*Note*: II: Interaction intelligence; IH: Interaction humanness; IM: Immersion; PSR: Parasocial relationships; SME: Social media engagement; DN: Donation; PI: Purchase intention.

**Table 4:** Heterotrait-Monotrait ratio of correlations (HTMT).

	SME	DN	IH	IM	II	PSR	PI
**SME**							
**DN**	0.786						
**IH**	0.408	0.556					
**IM**	0.737	0.668	0.462				
**II**	0.572	0.520	0.618	0.625			
**PSR**	0.806	0.711	0.600	0.727	0.628		
**PI**	0.737	0.713	0.465	0.644	0.519	0.696	

*Note*: II: Interaction intelligence; IH: Interaction humanness; IM: Immersion; PSR: Parasocial relationships; SME: Social media engagement; DN: Donation; PI: Purchase intention.

### 5.2. Common method variance and multicollinearity

In this study, we used two statistical methods to assess CMV. First, Harman’s one-factor test was implemented to address the potential CMB problem, revealing that the largest variance explained by a single factor was 43.314%, which is less than the recommended 50% threshold [118].

Secondly, following Liang et al. (2007) [119], we incorporated a common method factor into the model. Results showed that the average substantive constructs explained 70.65% of the variance, but the common method factor only explained 0.70% of the variance; the ratio between them was 100.93:1. In addition, most method factor loadings were not significant. These analyses suggest that CMB is not a problem for the current study.

Additionally, to address the multicollinearity problem, a full-collinearity test was conducted. The results show that full-collinearity VIF values ranged from 1.346 to 1.615 for all latent constructs, remaining well below the 3.3 threshold.

### 5.3. Structural model

We used the standardized root mean square residuals (SRMR) to assess model fit of the structural model [120,121]. The value of SRMR in this study was 0.069, meeting commonly accepted criteria (SRMR<0.100) and indicating a well-fitted model. The model accounted for 53% of the variance in social media engagement, 45.8% in donation, 44.3% in purchase intention, 48.0% in parasocial relationships, and 25.7% in immersion. In addition, this study also evaluated the Q^2^ values of the potential endogenous constructs in the model via the blindfold procedure. The results showed that the Q^2^ values of immersion (Q^2^ = 0.165), parasocial relationships (Q^2^ = 0.335), social media engagement (Q^2^ = 0.294), donation (Q^2^ = 0.372), and purchase intention (Q^2^ = 0.362) were all greater than zero, indicating predictive relevance for the endogenous constructs within the PLS-SEM blindfolding assessment.

As shown in Table 5 and Fig 2, interaction intelligence was significantly and positively associated with immersion (β = 0.389, p < 0.001) and parasocial relationships (β = 0.196, p < 0.001). H1a and H1b were supported. Interaction humanness was significantly and positively related to immersion (β = 0.177, p < 0.01) and parasocial relationships (β = 0.295, p < 0.001). H2a and H2b were supported. Immersion was significantly and positively associated with parasocial relationships (β = 0.371, p < 0.001), social media engagement (β = 0.275, p < 0.001), donation (β = 0.271, p < 0.001), and purchase intention (β = 0.261, p < 0.001). H3a-H3d were supported. Parasocial relationships were significantly and positively related to social media engagement (β = 0.530, p < 0.001), donation (β = 0.461, p < 0.001), and purchase intention (β = 0.441, p < 0.001). H4a-H4c were supported.

**Table 5:** Hypothesis results.

Num	Hypothesis	Coefficient	Std.Dev.	p-value	f^2^	Result
H1a	II - > IM	0.389	0.057	<.001	0.144	Support
H1b	II - > PSR	0.196	0.053	<.001	0.046	Support
H2a	IH - > IM	0.177	0.052	<.01	0.030	Support
H2b	IH - > PSR	0.295	0.052	<.001	0.115	Support
H3a	IM - > PSR	0.371	0.055	<.001	0.196	Support
H3b	IM - > SME	0.275	0.053	<.001	0.106	Support
H3c	IM - > DN	0.271	0.050	<.001	0.089	Support
H3d	IM - > PI	0.261	0.050	<.001	0.081	Support
H4a	PSR - > SME	0.530	0.052	<.001	0.381	Support
H4b	PSR - > DN	0.461	0.046	<.001	0.249	Support
H4c	PSR - > PI	0.441	0.067	<.001	0.223	Support

*Note*: II: Interaction intelligence; IH: Interaction humanness; IM: Immersion; PSR: Parasocial relationships; SME: Social media engagement; DN: Donation; PI: Purchase intention.

**Fig 2:**
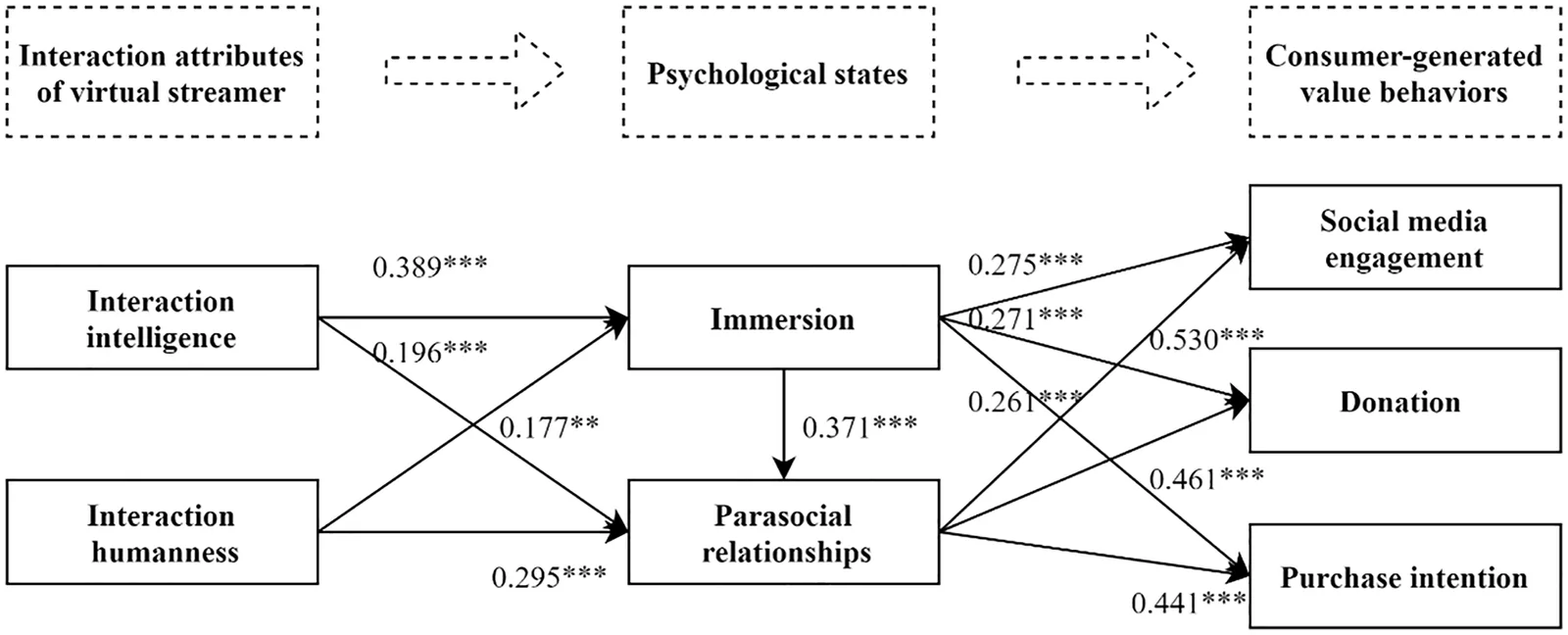
The results of the structural model.

In addition to assessing the significance of the path coefficients, we evaluated the effect sizes (f^2^) to determine the practical relevance of the supported structural associations. Following the guidelines by Cohen (1988) [122], f^2^ values of 0.02, 0.15, and 0.35 represent small, medium, and large effect sizes, respectively. As presented in Table 5, all supported paths demonstrated meaningful effect sizes ranging from 0.030 to 0.381. Notably, the structural association between parasocial relationships and social media engagement showed a large f²value (0.381), while several other supported paths showed medium or small but practically meaningful f²values.

Regarding control variables, we included age, sex, and education in the model. Results showed that sex was significantly associated with donation (β = −0.159, p < 0.05); male fans’ willingness to donate was higher than female fans’ (M male = 5.2655, M female = 4.6693, t (477) =4.500). Additionally, age was significant and positively associated with purchase intention (β = 0.110, p < 0.01). A specific breakdown of the group means shows that purchase intention was 4.82 for those aged ≤20, 5.10 for ages 21–24, 5.71 for ages 25–30, 5.77 for ages 31–35, 5.95 for ages 36–40, 5.22 for ages 41–50, and 5.94 for ages 51–60. These descriptive results suggest that purchase intention was generally higher among respondents aged 25–40 than among the two youngest groups, although this pattern should be interpreted cautiously because it is based on descriptive group means and the age distribution of the sample was uneven.

### 5.4. Sequential indirect associations

To further examine the sequential indirect associations linking virtual streamers’ interaction attributes, immersion, parasocial relationships, and consumer-generated value behaviors, this study adopted a bootstrapping method with 5,000 resamples. The results indicated that all of the sequential indirect associations were significant based on the bias-corrected confidence intervals (Table 6).

**Table 6:** Results of sequential indirect association tests.

Indirect association path	Indirect association estimate	95% BC-CI	Result
II - > IM - > SME	0.107	[0.059, 0.165]	Significant
II - > IM - > DN	0.106	[0.059, 0.158]	Significant
II - > IM - > PI	0.102	[0.055, 0.161]	Significant
II - > IM - > PSR	0.144	[0.090:0.205]	Significant
II - > IM - > PSR - > SME	0.076	[0.047, 0.110]	Significant
II - > IM - > PSR - > DN	0.066	[0.040, 0.099]	Significant
II - > IM - > PSR - > PI	0.064	[0.037, 0.096]	Significant
II - > PSR - > SME	0.104	[0.047, 0.165]	Significant
II - > PSR - > DN	0.090	[0.042, 0.139]	Significant
II - > PSR - > PI	0.086	[0.038, 0.142]	Significant
IH - > IM - > SME	0.049	[0.019, 0.087]	Significant
IH - > IM - > DN	0.048	[0.018, 0.085]	Significant
IH - > IM - > PI	0.046	[0.017, 0.085]	Significant
IH - > IM - > PSR	0.066	[0.026, 0.113]	Significant
IH - > IM - > PSR - > SME	0.035	[0.014, 0.059]	Significant
IH - > IM - > PSR - > DN	0.030	[0.012, 0.052]	Significant
IH - > IM - > PSR - > PI	0.029	[0.012, 0.051]	Significant
IH - > PSR - > SME	0.157	[0.095, 0.229]	Significant
IH - > PSR - > DN	0.136	[0.082, 0.202]	Significant
IH - > PSR - > PI	0.130	[0.071, 0.204]	Significant
IM - > PSR - > SME	0.196	[0.138, 0.259]	Significant
IM - > PSR - > DN	0.171	[0.116, 0.234]	Significant
IM - > PSR - > PI	0.163	[0.105, 0.225]	Significant

*Note*: II: Interaction intelligence; IH: Interaction humanness; IM: Immersion; PSR: Parasocial relationships; SME: Social media engagement; DN: Donation; PI: Purchase intention.

### 5.5. Robustness check: Competing model analysis

Although our primary specification models immersion and parasocial relationships in the IM → PSR structural ordering, we acknowledge that an alternative or reciprocal association between these constructs is theoretically plausible. Viewers who report feeling close to a streamer may also report higher engagement with, or immersion in, subsequent viewing experiences. Recent literature [123] also suggests that parasocial relationships are associated with cognitive absorption, a state closely related to immersion. Therefore, to assess the robustness of our proposed specification, we tested a competing model in which the focal structural ordering was changed from “immersion → parasocial relationships” to “parasocial relationships → immersion”. Because the data are cross-sectional and self-reported, this analysis was not intended to establish temporal or causal directionality, but rather to evaluate whether the proposed specification receives comparatively greater empirical support than a plausible alternative specification. Both models were compared in SmartPLS 4.0 using structural consistency, model fit, the Bayesian Information Criterion, BIC-based model weights, and predictive performance [114].

Overall, the comparison results provide comparatively greater support for the proposed model, although they do not rule out alternative structural orderings or reciprocal associations. In the alternative model, the structural path linking interaction humanness to immersion became non-significant (β = 0.017, p = 0.743), whereas this path was significant in the original model (β = 0.177, p < 0.01). This result does not imply that the alternative model breaks down, it suggests that the alternative specification is less consistent with the hypothesized association between interaction humanness and immersion. In terms of information criteria, BIC values and BIC-based model weights favored the original model for social media engagement, donation, immersion, and purchase intention, whereas the alternative model was favored for parasocial relationships. Notably, the stronger BIC-based support for the alternative model with respect to parasocial relationships suggests that the association between immersion and parasocial relationships may be more complex than a strictly unidirectional structural ordering and may involve reverse or reciprocal associations. However, the CVPAT results showed no significant difference in global predictive performance between the two models (p = 0.270), indicating that their overall predictive capability was comparable. The original model also showed a marginally better model fit than the alternative model (SRMR = 0.069 vs. 0.071). Taken together, these findings provide comparatively greater support for the proposed model, while also indicating that the alternative specification remains plausible. Given the cross-sectional and self-reported nature of the data, this analysis cannot establish temporal or causal directionality and does not rule out reverse or reciprocal associations between immersion and parasocial relationships. Detailed statistical comparisons are provided in Table A in S1 Appendix.

## 6. Implications

### 6.1. Discussion

This study explored the relationships between interaction attributes of virtual streamers and consumer psychology and behavior. The results indicate that both interaction intelligence and interaction humanness are positively related to consumer immersion and parasocial relationships. Furthermore, immersion is positively associated with parasocial relationships. Ultimately, both immersion and parasocial relationships are positively associated with higher levels of purchase intention, social media engagement, and donation.

(1) Interaction intelligence and interaction humanness are positively associated with immersion

Our findings reveal that the two interaction attributes of virtual streamers are associated with stronger immersive experiences. Building upon prior research indicating that stronger interaction is associated with stronger immersion [56,57,84], this study extends the literature by distinguishing interaction intelligence from interaction humanness and showing that both are significantly associated with consumer immersion. This suggests that virtual streamers’ ability to respond intelligently and display human-like qualities is related to stronger psychological involvement and real-time experience among consumers. The finding enriches the understanding of interaction in virtual environments by highlighting the importance of interaction quality and conceptualization, rather than only frequency or functional capability [37,39]. The analysis further shows that interaction intelligence has a stronger association with immersion than interaction humanness. This suggests that, in the early stages of virtual interaction, viewers tend to report stronger immersion when they perceive intelligent and highly relevant interaction performance. Such responsiveness is associated with a smoother interaction state, stronger attention, and more favorable real-time experience among consumers. Conversely, interaction humanness appears to be more closely related to the emotional dimension, as well as to the continuity and stability of immersion. This implies that technical optimization may be particularly relevant to stronger immersive experiences, while emotional and intentional expression may be related to the maintenance of immersion quality.

(2) Interaction intelligence and interaction humanness are positively associated with parasocial relationships

Interaction has been regarded as an important factor related to maintaining relationships in online environments [89] and has been associated with parasocial relationship formation [90,124]. This study finds that both interaction intelligence and interaction humanness are positively associated with parasocial relationships, aligning with previous work showing that parasocial relationships are stronger in contexts of repeated engagement with the same media character [30]. Prior studies have also shown that virtual streamers’ comments, greetings, and real-time responses are associated with stronger psychological connections with viewers, as well as with trust and positive relationships [4,33,91]. Building on this, the study shows that both interaction attributes are significantly and positively associated with parasocial relationships. Interaction intelligence is associated with viewers’ perceptions of the streamer’s understanding capability, while interaction humanness is associated with a stronger sense of self-will and social presence, as well as consumers’ tendency to view the streamer as a quasi-human social partner rather than a technological entity. This quasi-social interaction is associated with stronger emotional projection and long-term attachment, as well as stronger parasocial bonds [19].

Notably, interaction humanness showed a stronger association with parasocial relationships than interaction intelligence. This suggests that consumers not only want virtual streamers to understand them, but also to possess independent thoughts and intentions. When streamers display such qualities, viewers tend to report stronger perceptions of them as social others with personality and consciousness, as well as stronger quasi-social emotional bonds similar to those associated with human streamers or idols [4,125]. Thus, interaction humanness is not only related to emotional expression but also associated with stronger perceived existential meaning of virtual streamers, extending parasocial relationship theory by showing that human-machine relationships can approach interpersonal relationships.

(3) Immersion is positively associated with parasocial relationships

Previous research has rarely examined the link between immersion and parasocial relationships. This study shows that immersion is significantly and positively associated with parasocial relationships. When viewers become deeply immersed in a virtual streamer’s livestream, their cognitive focus and emotional investment tend to be directed toward the streamer, which is associated with lower awareness of the medium and stronger perceptions of the streamer as a real social entity. This immersive state is closely related to emotional attachment. This result further indicates that immersion is not merely related to technical presentation or focused attention, but also represents a psychological state with social and emotional qualities [126]. When virtual streamers display both logical intelligence and human-like intentions, consumers tend to report a stronger sense that they are communicating with a conscious being, which is associated with emotional bonding. Crucially, our findings highlight that in the relationship formation stage, the episodic state of immersion is an important psychological state associated with enduring parasocial bonds.

(4) The sequential indirect associations of immersion and parasocial relationships

Previous studies have examined the relationship between interaction and immersion [55,84] and relationship building [89,124], as well as the associations of immersion and parasocial relationships with behaviors such as purchase intention and donation [56,57,80,108]. In the context of virtual streamers, this study identifies the sequential indirect associations involving immersion and parasocial relationships in the relationships between interaction intelligence, interaction humanness, and consumer behavior. Immersion appears to be more closely related to cognitive engagement, while parasocial relationships appear to be more closely related to emotional connection, together representing a sequential psychological association pathway between interaction attributes and behavioral responses. In addition, immersion and parasocial relationships show stronger associations with social media engagement than with purchase intention and donation. This suggests that virtual streamers’ interaction attributes are more strongly associated with emotional and social participation than with immediate economic consumption. The value creation pattern of virtual streamers therefore appears to be closely related to emotional co-creation and social interaction, whereas economic behaviors may be more closely associated with the accumulation of these psychological processes and the support of additional material or practical incentives [53,127].

### 6.2. Theoretical implications

(1) Conceptualizing the intelligence-humanness duality of the virtual streamer interaction

This study refines the conceptualization of “interaction” within the virtual streamer research. Prior studies have primarily focused on general streamer characteristics (e.g., attractiveness [13], overall anthropomorphism [11,21]) or singular interactive dimensions, such as frequency and responsiveness [4,12,33]. Such approaches often overlook the multifaceted nature of virtual interactions. We address this gap by deconstructing this construct into two core dimensions: interaction intelligence (the capacity to understand and respond) and interaction humanness (the manifestation of empathy and thoughtfulness). Grounded in human-computer interaction literature, this dual-dimension framework illustrates how distinct interaction traits are associated with specific psychological responses, enabling researchers to examine these relationships more precisely.

(2) Linking immersion and parasocial relationships in a sequential psychological pathway

This research links immersion theory [16,17] with parasocial relationship theory [18–20] to explain the psychological association patterns in virtual human-computer interactions. While previous literature typically examines immersion and parasocial relationships in isolation [56,59,69,128], our study identifies a sequential indirect association pattern. Specifically, the intelligence and humanness of virtual streamers are associated with consumers’ immersive experiences, which are further associated with parasocial relationships. By illustrating how immersion is linked to both cognitive engagement and emotional connection, these findings advance both theoretical streams. We show that parasocial relationship formation is not only associated with surface-level traits [63,64] or technological affordances [65], but is also sequentially associated with the state of immersion related to intelligent and human-like interactions.

(3) Advancing the literature on consumer-generated value behaviors

This study broadens the scope of consumer value creation in digital environments. Existing live-streaming research often treats user actions (e.g., purchasing, donating, and social media engagement) as isolated dependent variables, with a heavy emphasis on purchase intention [10,12,33,53]. This fragmented approach overlooks the shared psychological factors associated with these co-creation activities. Drawing on previous literature [77], we integrate purchase intention, social media engagement, and donation into a unified framework: consumer-generated value behaviors. By empirically demonstrating how interaction intelligence and humanness are positively associated with these behavioral outcomes through sequential psychological association pathways, our study highlights the strategic importance of deep virtual interactions in relation to multifaceted value co-creation.

### 6.3. Practical implications

(1) Enhancing virtual streamer interaction attributes to support diversified consumer-generated value behaviors

The results indicate that interaction intelligence and interaction humanness are positively associated with consumer immersion and parasocial relationships, which are further associated with multidimensional consumer-generated value behaviors such as purchase intention, donation, and social media engagement. Therefore, virtual streamer designers and operators should focus on improving the quality, response speed, and semantic relevance of virtual streamer interactions. From a technical perspective, advanced natural language processing and sentiment recognition technologies can be optimized to support virtual streamers’ prompt and accurate responses to viewers’ questions. Simultaneously, virtual streamers should demonstrate their own thoughts and emotions during interactions. Such high-quality interactions may be associated with stronger consumer immersion, closer parasocial bonds, and more diverse consumer-generated value behaviors.

(2) Strategies and practices for enhancing consumer immersive experiences

This study emphasizes the importance of immersion as a psychological state associated with the relationship between virtual streamer interaction attributes and consumer behavior. Immersion is associated with consumers’ sense of mentally “entering” the live streaming context, experiencing the virtual streamer’s authentic presence and emotional investment, and reporting deeper experiential involvement. In practice, the content and scene design of virtual streamers should focus on enhancing immersion quality. For instance, multimodal sensory design (such as the coordination of visual, auditory, and verbal feedback), immersive storytelling, and gamified interaction mechanisms may support consumers’ sense of presence and sustained engagement. Additionally, maintaining the coherence and rhythm of interactive content may be associated with sustained attention and emotional connection, as well as with stronger immersion and subsequent value-generating behaviors.

(3) Actively building parasocial relationships between virtual streamers and consumers

Given that parasocial relationships are involved in the indirect association between virtual streamer interaction attributes and consumer-generated value behaviors, consumers’ emotional attachment and psychological closeness to virtual streamers are associated with their behavioral intentions. Therefore, platforms and developers should focus on designing interactions characterized by social warmth, familiarity, and emotional reciprocity. For example, maintaining consistency in the virtual streamer’s image and persona, offering personalized responses during interactions, and appropriately displaying “self-disclosure” (such as expressing feelings and showing attitudes) may be associated with stronger consumers’ psychological connection. Strong parasocial relationships are not only associated with greater consumer loyalty and repeat purchase intention but also related to sustained interaction and word-of-mouth, which may support the long-term maintenance of consumer relationships.

### 6.4. Limitations and future research

Despite our theoretical and practical contributions, several limitations of this study present opportunities for future research. First, the data were collected within a single cultural context, potentially limiting the generalizability of the findings. Subsequent research should examine whether the observed association patterns of interaction intelligence and humanness remain consistent across diverse cultural settings. Second, while this study isolated the core interaction attributes of virtual streamers, these digital personas are highly diverse media. Specific characteristics, such as gender, persona setting, and avatar aesthetics, may be associated with varying responses among different audience segments. Incorporating these personalized factors into the analytical framework would provide a more nuanced understanding of consumer behavior in this domain. Third, our reliance on cross-sectional, self-reported survey data presents inherent methodological limitations. Because all constructs were measured from the same respondents at a single point in time, we cannot completely rule out common method bias, despite the acceptable results from our post hoc statistical checks (e.g., Harman’s one-factor test and the common method factor). Consequently, this study can only establish predictive associations rather than strict causal relationships. While our findings and robustness checks are consistent with the proposed ordering from immersion to parasocial relationships, which captures the relationship formation stage, we acknowledge that a reciprocal loop is plausible during the relationship maintenance stage. Specifically, established fans with high parasocial relationships might naturally experience deeper immersion in subsequent viewing sessions [123]. Future longitudinal or experimental designs could examine this reciprocal spiral between episodic immersion and cumulative relational bonds over time. Finally, although immersion is specified as a primary mediating variable in our model, its relatively lower explained variance suggests the presence of unmodeled correlates. We focused on the streamer’s interaction attributes as key associated factors, but immersion is a complex phenomenon. It is likely associated with technological factors (e.g., rendering quality, real-time latency), environmental cues (e.g., the social atmosphere of live comments), and individual traits (e.g., trait absorption). Future studies are encouraged to integrate these variables to develop a more comprehensive network of associations for immersion in virtual live streaming.

## Supporting information

S1 Appendix Comparison between the original model and alternative model. (PDF)

S1 File Data. (XLSX)
