# Supplementary material for: Unveiling the role of virtual streamer’s interaction attributes in shaping consumer-generated value behaviors
Source: PLoS One. 2026 Nov 11;21(11):e0361335. doi: 10.1371/journal.pone.0361335 (PMC13754118; doi:10.1371/journal.pone.0361335)
Supplement: Supplementary file 1 [file pone.0361335.s001.pdf]

Appendix

Table A. Comparison between the original model and alternative model.

Evaluation Criteria	Metric / Construct	Model A: Original Model (IM → PSR)	Model B: Alternative Model (PSR → IM)	Conclusion
Structural Integrity	Path: IH → IM	β = 0.177 (p < 0.01)	β = 0.017 (p = 0.743)	The alternative model is less consistent with the hypothesized association between IH and IM.
	Path: IM →PSR	β = 0.371 (p < 0.001)	–	
	Path: PSR → IM	–	β = 0.443 (p < 0.001)	The PSR–IM association is statistically significant in the alternative specification, but temporal or causal directionality cannot be

Evaluation Criteria	Metric / Construct	Model A: Original Model (IM → PSR)	Model B: Alternative Model (PSR → IM)	Conclusion
				established with cross-sectional data.
Model Fit	SRMR	0.069	0.071	The original model shows marginally better fit.
Information Criteria	BIC / BIC-based Model Weight: SME	BIC = -329.744; weight = 0.887	BIC = -325.631; weight = 0.113	The original model receives stronger relative statistical support for SME.
	BIC / BIC-based Model Weight: DN	BIC = -263.175; weight = 0.951	BIC = -257.228; weight = 0.049	The original model receives stronger relative statistical support for DN.
	BIC / BIC-based Model Weight:	BIC = -204.476; weight	BIC = -124.790; weight	The original model receives stronger relative

Evaluation Criteria	Metric / Construct	Model A: Original Model (IM → PSR)	Model B: Alternative Model (PSR → IM)	Conclusion
	IM	= 1.000	= 0.000	statistical support for IM.
	BIC / BIC-based Model Weight: PSR	BIC = -210.026; weight = 0.000	BIC = -289.525; weight = 1.000	The alternative model receives stronger relative statistical support for PSR.
	BIC / BIC-based Model Weight: PI	BIC = -249.252; weight = 0.910	BIC = -244.617; weight = 0.090	The original model receives stronger relative statistical support for PI.
Predictive Validity	CVPAT (Global Model)	1.249	1.246	No significant difference in global predictive performance (p=0.270).

Note: IH = Interaction Humanness; IM = Immersion; PSR = Parasocial Relationships; SME = Social Media Engagement; DN = Donation; PI = Purchase Intention. BIC-based

model weights are normalized weights calculated from Bayesian Information Criterion values; lower BIC values indicate better relative model performance, and higher BIC-based weights indicate stronger relative support among the compared model specifications.
